# An exploratory study of cell stiffness as a mechanical label-free biomarker across multiple musculoskeletal sarcoma cells

**DOI:** 10.1186/s12885-023-11375-3

**Published:** 2023-09-12

**Authors:** Cyril Daniel, Frank Traub, Saskia Sachsenmaier, Rosa Riester, Moritz Mederake, Christian Konrads, Marina Danalache

**Affiliations:** 1 Laboratory of Cell Biology, Department of Orthopedic Surgery, University Hospital of Tübingen, 72076 Tübingen, Germany; 2 Department of Orthopedic Surgery, University Hospital of Tübingen, 72076 Tübingen, Germany; 3 Department of Orthopedics and Traumatology, University Medical Center Mainz, Johannes Gutenberg-University Mainz, 55122 Mainz, Germany; 4 Department of Trauma and Reconstructive Surgery, BG Clinic, University of Tübingen, 72076 Tübingen, Germany; 5 Department of Orthopedics and Traumatology, Hanseatic Hospital Stralsund, 18437 Stralsund, Germany

**Keywords:** Cancer, Musculoskeletal sarcomas, Atomic force microscopy, Stiffness, Biomarker, Cytoskeleton

## Abstract

**Background:**

Cancer cells are characterized by changes in cell cytoskeletal architecture and stiffness. Despite advances in understanding the molecular mechanisms of musculoskeletal cancers, the corresponding cellular mechanical properties remain largely unexplored. The aim of this study was to investigate the changes in cellular stiffness and the associated cytoskeleton configuration alterations in various musculoskeletal cancer cells.

**Methods:**

Cell lines from five main sarcoma types of the musculoskeletal system (chondrosarcoma, osteosarcoma, Ewing sarcoma, fibrosarcoma and rhabdomyosarcoma) as well as their healthy cell counterparts (chondrocytes, osteoblasts, mesenchymal stem cells, fibroblasts, skeletal muscle cells) were subjected to cell stiffness measurements via atomic force microscopy (AFM). Biochemical and structural changes of the cytoskeleton (F-actin, β-tubulin and actin-related protein 2/3) were assessed by means of fluorescence labelling, ELISA and qPCR.

**Results:**

While AFM stiffness measurements showed that the majority of cancer cells (osteosarcoma, Ewing sarcoma, fibrosarcoma and rhabdomyosarcoma) were significantly less stiff than their corresponding non-malignant counterparts (p < 0.001), the chondrosarcoma cells were significant stiffer than the chondrocytes (p < 0.001). Microscopically, the distribution of F-actin differed between malignant entities and healthy counterparts: the organisation in well aligned stress fibers was disrupted in cancer cell lines and the proteins was mainly concentrated at the periphery of the cell, whereas β-tubulin had a predominantly perinuclear localization. While the F-actin content was lower in cancer cells, particularly Ewing sarcoma (p = 0.018) and Fibrosarcoma (p = 0.023), this effect was even more pronounced in the case of β-tubulin for all cancer-healthy cell duos. Interestingly, chondrosarcoma cells were characterized by a significant upregulation of β-tubulin gene expression (p = 0.005) and protein amount (p = 0.032).

**Conclusion:**

Modifications in cellular stiffness, along with structural and compositional cytoskeleton rearrangement, constitute typical features of sarcomas cells, when compared to their healthy counterpart. Notably, whereas a decrease in stiffness is typically a feature of malignant entities, chondrosarcoma cells were stiffer than chondrocytes, with chondrosarcoma cells exhibiting a significantly upregulated β-tubulin expression. Each Sarcoma entity may have his own cellular-stiffness and cytoskeleton organisation/composition fingerprint, which in turn may be exploited for diagnostic or therapeutic purposes.

## Introduction

Cancer cells differ from normal cells and these differences refer to alterations in cell morphology, cell-cell and cell-extracellular matrix (ECM) interactions, cell invasion and adhesion, as well as cell death [1]. Sarcomas of the musculoskeletal system are a heterogeneous group of malignant tumors, including over 80 different histological diagnoses [2]. Commonly, sarcomas are categorized into two main categories: bone sarcomas and soft tissue sarcomas. With diagnostic means getting better constantly, more and more subtypes are characterized, thus, steadily increasing the number of possible different diagnoses [3]. The majority of sarcoma subtypes continue to pose a significant diagnostic and treatment challenge, with overall survival rates falling between 20 and 30% [4]. Due to the high degree of heterogeneity in their genetic profile, histology, as well as clinical features, the implementation of specific biomarkers, could function as a diagnosis and therapeutic Achilles’ heel [5].

Cell stiffness has previously been proposed to serve as a label-free biomarker for cancer detection [6–8], and refers to a cell’s ability to deform in response to external stress. It has recently been emphasized that alterations in the mechanical properties of cells and the surrounding environment (i.e. extracellular matrix (ECM)) play a decisive role during malignant transformation and cancer progression [9]. In fact, several studies have shown that cells derived from primary tumors as well as metastatic cells (e.g., lung, breast, and pancreatic neoplasia) are more elastic than their benign counterparts [6, 10]. Such changes in cellular stiffness may be a feature of carcinogenesis or even part of cancer cell’s survival strategy to adapt to new environments. The cytoskeleton is the cell’s primary mechanical structure; it is a complex, dynamic biopolymer network composed of microtubules, actin, and intermediate filaments [11]. Actin filaments (F-actin) along with multi-protein actin complexes (i.e. ARPC2/3) dictate the directionality, orientation, and large-scale architecture of the cytoskeleton framework [12], and are thought to be major contributors to cell stiffness [13]. The close link between actin filaments and cell stiffness is well documented by the use of disruptive pharmacological agents such as cytochalasin D [14]. Microtubules, like actin, play also an important role, with magnetic twisting cytometry studies showing that destabilizing microtubules decreased cell stiffness while stabilizing them increased it [15].

The advancement of technology in measuring stiffness of individual cells has resulted in powerful techniques capable of bridging the gap between mechanical properties and cellular functioning and structures. Pioneering research over two decades ago demonstrated the importance of mechanical properties in characterizing cancerous cells [16]. Various techniques for probing the mechanics of tumors have been developed, with atomic force microscopy (AFM) emerging as an excellent platform for simultaneously characterizing the structures and mechanical properties of living biological systems [17]. It has a phenomenal spatiotemporal resolution, opening up new avenues for understanding tumor physics and contributing significantly to cancer research. Indeed, AFM-stiffness assessment has been demonstrated to be useful not only for early cancer diagnosis by measuring cancer-specific proteins, but also for cancer progression monitoring by correlating the amount of cancer-specific proteins with cancer progression [18]. This seems logical given that metastatic cells require the ability to deform in order to metastasize and infiltrate, and several studies have shown that cells with higher elasticity have increased invasiveness and metastatic potential [19, 20]. Cell mechanical properties in mammalian cells are primarily determined by the cell cytoskeleton network, where the density and arrangement of filaments, the number of cross-links, activity, and stress generation all influence elastic properties [21–23].

The aim of this study was to investigate the mechanical characteristic alterations (i.e. stiffness) that occur at the cellular level of a wide range of tissue sarcoma types (i.e. chondrosarcoma, osteosarcoma, fibrosarcoma, Ewing sarcoma, rhabdomyosarcoma) as well as their healthy counterparts and to gain insight into the cytoskeleton changes (actin and microtubules), that may cause such changes.

## Materials and methods

### Cell lines and culture

Five different sarcoma cell lines: chondrosarcoma (SW1353, #HTB-94, American Type Culture Collection (ATCC), Manassas, Virginia, USA)), osteosarcoma (SaOs-2, #HTB-85, ATCC), Ewing sarcoma (RD-ES, #HTB-166, ATCC), fibrosarcoma (HT-1080, #CCL-121, ATCC) and rhabdomyosarcoma (RD, #CCL-136, ATCC) were used in this study. SW1353 and SaOs-2 cells were cultured in Dulbecco’s Modified Eagle Medium DMEM/F12 (Gibco, Darmstadt, Germany) with 5% (v/v) FCS (fetal bovine serum; Gibco) and 1% (v/v) penicillin/streptomycin (Biochrom, Berlin, Germany), RD-ES cells in RPMI-1640 with L-glutamine (Gibco) media, supplemented with 15% (v/v) FCS (Gibco) and 1% (v/v) penicillin/streptomycin (Biochrom), while HT-1080 cells and RD were cultured in DMEM with GlutaMAX, 4.5 g/l D-glucose (Gibco) supplemented with 10% (v/v) FCS (Gibco) and 1% (v/v) penicillin/streptomycin (Biochrom). Chondrocytes isolated from femoral condyles collected from patients undergoing total knee arthroplasty were used as healthy control cells (for comparison with chondrosarcoma). As a control group for the osteosarcoma, human bone marrow- mesenchymal stem cells (MSC) were isolated, differentiated and propagated into osteoblasts as previously described [24]. Adult human fibroblasts (#PCS-201-012, ATCC) were used as a control for the fibrosarcoma, MSC for the Ewing sarcoma and primary human skeletal muscle cells (SKMC, #PCS-950-010, ATCC) for the rhabdomyosarcoma. Chondrocytes, fibroblasts, skeletal muscle cells were cultured in RPMI 1640 with L-glutamine (Gibco) supplemented with 10% (v/v) FCS with 1% (v/v) penicillin/streptomycin (Biochrom), while MSC were cultured in DMEM (Gibco) media containing 10% (v/v) human platelet lysate (hPL) and 1% (v/v) amphotericin B and penicillin/streptomycin (Biochrom), 200mM glutamine (Sigma-Adrich, St. Louis, Missouri, USA), 1 IU heparin (AppliChem GmbH, Darmstadt, Germany). The hPL was purchased from the Tübingen Centre for Clinical Transfusion Medicine; it did not contain heparin and was referred to as a research lysate due to the absence of a quarantine period. All cells were cultured at 37 °C in the incubator supplied with 5% CO_2_ and trypsin-versene EDTA (1X, Lonza, Basel, Switzerland) was used to passage cells.

### Cell stiffness assessment - atomic force microscopy

**Fig. 1:**
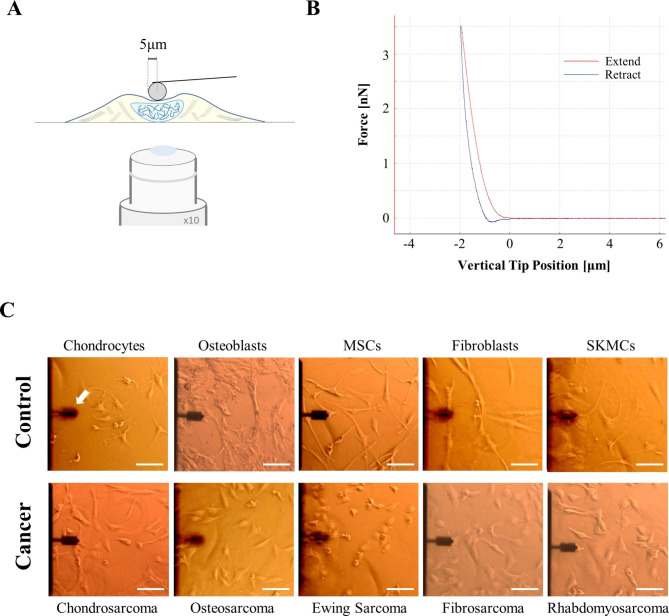
Experimental settings for cell stiffness assessment. (**A**) Schematic representation of cell micro-indentations using a 5 μm radius spherical cantilever. (**B**) Representative force-distance curve obtained from AFM indentations for cells. The extend curve that is used for fitting of the Hertz fit model is shown in red. (**C**) Microscopic pictures of AFM measured cancer cell lines and their corresponding non-malignant counterparts. The cantilever used for measurements is also shown (white arrow). Scale bar (white) represents 100 μm. Abbreviations: AFM – atomic force microscopy

All cell types were seeded at a density of 3 × 10^4^ in petri dishes (TPP Techno Plastic Products AG, Trasadingen, Switzerland) and covered with Leibovitz’s L-15 medium w/o l-glutamine (Merck KGaA, Darmstadt, Germany) media. The stiffness of living cells was assessed using a CellHesion200 (Bruker, Billerica, Massachusetts, USA) atomic force microscope (AFM) system, mounted onto an inverted light microscope (AxioObserver D1, Carl Zeiss Microscopy, Jena, Germany). A 5 μm radius spherical tip (model: SAA-SPH-5 μm, k = 0.2 N/m, Bruker) was used for microscale indentation (Fig. 1 - A). The cantilever was calibrated on the extended curve, and the spring constant was determined using the thermal noise method built into the device software (Bruker, Fig. 1 - B). In force spectroscopy mode, force-distance curves were sampled at 2 kHz, with a force trigger of 3nN and a velocity of 2 μm/sec. To assess the stiffness of the cells, we performed indentations on selected cells identified by microscopic examination (3 repetitions/cell; 90 cells per each cancer entity, Fig. 1 - C). Using the Hertz-fit model for spherical intenders included in the data processing software (Version 5.0.86, Bruker), the cell stiffness in the form of the Young’s modulus or elastic modulus (EM) was calculated from the force-distance curves. The Hertzian model’s equations are shown in Eq. 1 and Eq. 2.

1 $$F=\frac{E}{1-{v}^{2}} \left[\frac{{a}^{2}+{R}_{s}^{2}}{2}ln\frac{{R}_{s}+a}{{R}_{s}-a}-a{R}_{s}\right]$$

2 $$\delta =\frac{a}{2}ln\frac{{R}_{s}+a}{{R}_{s}-a}$$

Where *F* = Force; *E* = Young’s Modulus; *v* = Poisson’s ratio (which was set at 0.5); δ = indentation; *a* = radius of contact circle; *R*_*s*_ = radius of the sphere.

### Cytoskeleton structural investigation – immunolabelling

Following AFM measurements, the cell lines were labeled with F-actin and β-tubulin. Briefly, the cells were fixed for 10 min with 4% (v/v) paraformaldehyde (PFA, Sigma-Adrich) in PBS at room temperature and then washed three times with PBS. For F-actin, a solution of 1μM CellMask™ Green Actin Tracking Stain (#A57243; Thermo Scientific, Waltham, Massachusetts, USA) in 1% (w/v) bovine serum albumin (BSA) in PBS was used for 1 h. For β-tubulin, the cells were then incubated with anti- β-tubulin (rabbit, 1:100, #2129 9F3, Cell Signaling, Danvers, MA, United States) in 1% (w/v) BSA-PBS overnight at 4 °C in a humidity chamber. Afterward, the cells were washed three times with PBS and incubated with a secondary conjugated antibody (Alexa Fluor-594 goat anti-rabbit IgG, #a21429, Thermo Scientific) at a dilution of 1:200 in 1% (w/v) BSA-PBS for 2 h at room temperature in the dark. Nuclear staining was performed with 2 μg/ml DAPI (4′,6-diamidino-2-phenylindole, Thermo Fisher Scientific). The cells were washed three times for 5 min each in PBS. Images were acquired using a Leica DMi8 microscope (Leica, Wetzlar, Germany) at a 40x objective.

### Cytoskeleton biochemical investigation - ELISA

The changes in F-actin and β-tubulin content were analyzed by means of enzyme-linked immunosorbent assay (ELISA). For protein isolation, cells (density 1 × 10^6^) were washed with PBS and lysed in protein lysis buffer (40 mM Tris/HCl pH 7.4, 300 mM NaCl, 2 mM EDTA, 20% (v/v) glycerol, 2% (v/v) Triton X-100) supplemented with 1X proteinase inhibitor (Halt™ Protease-Inhibitor-Cocktail, Thermo Scientific) at 4 °C. A soluble fraction was obtained by centrifugation at 15,000* g* for 15 min at 4° C. Protein aliquots were first tested for total protein concentration using the Bradford protein assay before being normalized (Bio-Rad Laboratories, Richmond, CA, USA). A total of 20 μg of protein for each cell type was subjected to F-actin ELISA (#CSB-E13678h, Cusabio Technology LLC, Houston, Texas, USA) and β-tubulin ELISA (#RD-TuBb1-Hu, Reddot Biotech, Kelowna, Canada) following the manufacturer’s protocol. Absorbance was recorded at 450 nm by using an EL 800 reader (BioTek Instruments GmbH, Bad Friedrichshall, Germany). Three independent measurements of the ELISA assays were performed for each cell type.

### Cytoskeleton gene analysis

RNA was isolated from the cells (1 × 10^6^) using the RNeasy Mini kit (Qiagen, Hilden, Germany). Using the innuSCRIPT reverse transcriptase (Analytik Jena, Jena, Germany), 1 μg of RNA was reverse transcribed. A NanoDrop™ (Thermo Scientific) spectrophotometer was used to determine the purity and concentration of RNA. In a total volume of 10 μl, cDNA (50ng) was analyzed in duplicate reactions by quantitative-real-time-PCR (qRT-PCR) using gene-specific primers and 1X SYBR select master mix for CFX (Life Technologies GmbH). Primer pairs: F-actin (for: 5´-ACAGAGCCTCGCCTTTG-3´, rev: 5´-CCTTGCACATGCCGGAG-3´), β-tubulin (for: 5´-TCTTGCCCCATACATACCTTG-3´,rev: 5´-TCACTGATCACCTCCCAGAA-3´), ARPC2/3 complex (for: 5´-CTCTGGAGCTGAAAGACACA-3´, rev: 5´-AGGTTGATGGTGTTGTCTCG-3´), were purchased from Eurofins Genomics (Ebersberg, Germany). qRT-PCR was carried out in a QuantStudio3 (Thermo Scientific) and was analyzed using data analysis software incorporated in the device (Thermo Scientific). Relative expression levels were calculated using the ΔΔCt (2^−ΔΔCt^) method as previously described [25], using GAPDH as reference gene.

### Statistical analysis

The data is either graphically displayed as the median in a boxplot, or as mean with standard deviation (SD) in a bar diagram. Normality of the data was assessed by histograms and Shapiro-Wilk test. Depending on the normality, the comparison between experimental results was performed either by a Kruskal-Wallis with Mann-Whitney-U test as a post hoc test (AFM data) or t-test as a post hoc test (ELISA and qRT-PCR). Statistical analysis was performed with SPSS Statistics 22 (version 280.0.0.0 (190), IBM Corp., Armonk, NY, USA).

## Results

**Fig. 2:**
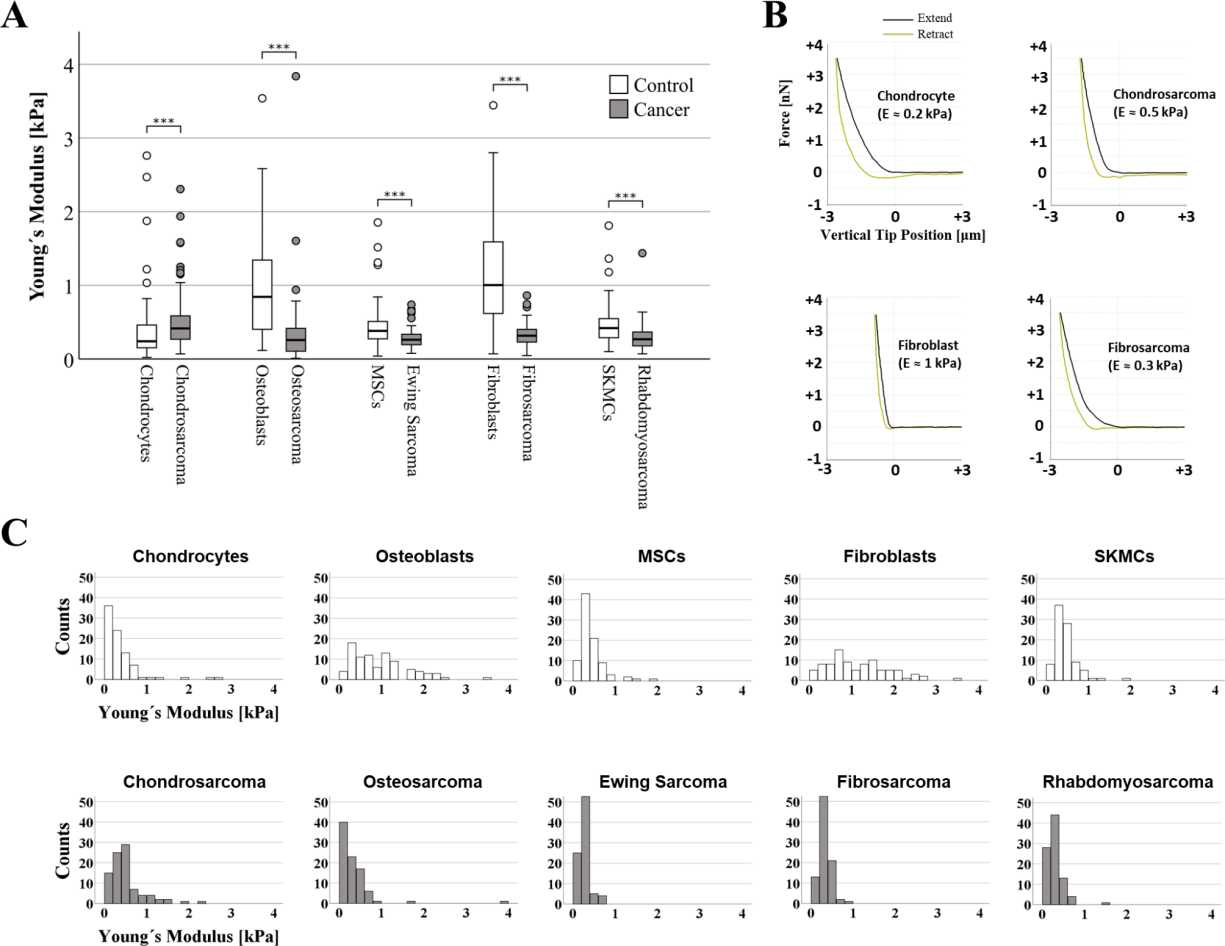
Analysis of Young’s modulus of musculoskeletal cancer cell lines and heathy controls. (**A**) Box plots (medians, first and third quartiles, minimum, maximum) of the cellular stiffness (kPa) for each cell line is displayed. Outliers are depicted by circles. Healthy control cells were stiffer than their corresponding neoplastic cell line (p < 0.001), only exception being the chondrocytes that exhibited a lower stiffness than the chondrosarcoma cell line (p < 0.001). (**B**) Representative force-distance curves for various cell types. (**C**) Images showing histogram distribution of elastic modulus of all cell types.*** p < 0.001. Abbreviations: MSC- mesenchymal stem cells, SKMC - skeletal muscle cells

For each cell type, 270 AFM measurements were conducted (n = 3 biological replicates, 30 cells/ replicate, 3 repetitions/ cell) and Fig. 2 displays the computed Young’s moduli. Cancer cells were significantly less stiff (more elastic) than their corresponding non-malignant counterparts (osteosarcoma cells - osteoblasts: p < 0.001, Ewing sarcoma cells - MSC: p < 0.001, fibrosarcoma cells - fibroblasts: p < 0.001, rhabdomyosarcoma cells - SKMC: p < 0.001) with the exception of chondrosarcoma cells and chondrocytes, where the trend was the opposite (p < 0.001, Fig. 2). Absolute values were thereby reduced by 69% for the osteoblast - osteosarcoma group (median: 0.842 kPa to 0.256 kPa), 31% for the MSC - Ewing sarcoma (median: 0.381 kPa to 0.260 kPa), 70% for the fibroblasts - fibrosarcoma (median: 1.008 kPa to 0.315 kPa) and 36% for the SKMC- rhabdomyosarcoma group (median: 0.418 kPa to 0.267 kPa), while for the chondrocyte - chondrosarcoma group a notable increase in stiffness (i.e. a lower elastic profile) was observed (median: 0.239 kPa to 0.414 kPa corresponding to a 73% increase). In terms of stiffness distribution, malignant cells showed a unimodal skewed to the left distribution, while for normal cells the distribution is broader and have a higher stiffness. The chondrosarcoma cells exhibited a bimodal stiffness distribution with two prominent peaks at 0.29 ± 0.05 kPa (‘peak 1’) and 0.48 ± 0.06 kPa (‘peak 2’).

**Fig. 3:**
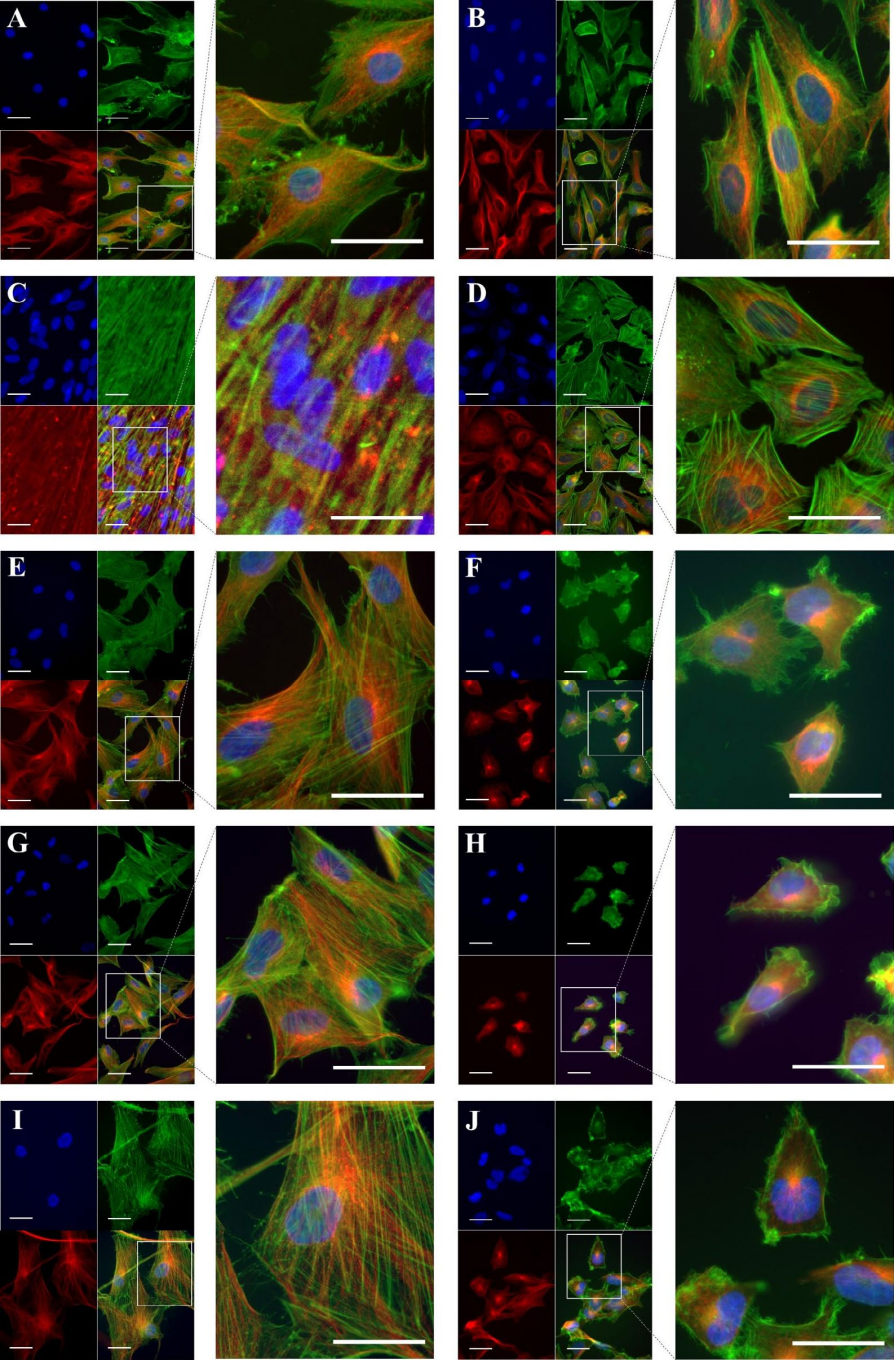
Cytoskeleton structure in musculoskeletal cancer lines and healthy cells. Healthy cells: (**A**) chondrocytes, (**C**) osteoblasts, (**E**) MSC, (**G**) fibroblasts, (**I**) SKMC and their corresponding cancer cell line: (**B**) chondrosarcoma, (**D**) osteosarcoma, (**F**) Ewing sarcoma, (**H**) fibrosarcoma and (**J**) rhabdomyosarcoma were subjected to β-tubulin immunolabelling (red) coupled with fluorescence labeling of F-actin (green). Cell nuclei are depicted in blue. Pictures taken at a 40x objective and scale bars (white) represent 50 μm. Abbreviations: MSC - mesenchymal stem cells, SKMC - skeletal muscle cells

Since cytoskeletal remodeling may be a major contributor to the observed differences in cell stiffness [26] between cancer cells and their healthy counterparts, we examined the cytoskeletal structure by F-actin and β-tubulin labelling. The results presented in Fig. 3A-J show that in comparison to all cancer cells (Fig. 3 - B, D, F, H, J), healthy cells show denser, better-aligned F-actin with longer stress fibers. Chondrocytes (Fig. 3 - A) showed a similar actin pattern, however at a lower density. For the stiffer healthy cells (Fig. 3 - C, E, G, I) the actin filaments are dispersed throughout the cell body, with actin bundles aligned along the long axis of the cell with well-defined stress fibers. Actin filaments in the softer cancer cells, in contrast, are less organized and F-actin bundles are oriented randomly with short segments, forming a tangled network (Fig. 3 - D, F, H, J). All cancer cells showed a predominant cortical F-actin structure, with the majority of filament lying in the cell’s periphery (Fig. 3 - B, D, F, H, J).

All of the cancer cell lines- healthy controls duo had a strong perinuclear presence of β-tubulin, forming a tortuous microtubular structure with longitudinally and obliquely concentrated crossed filaments, with a decreasing tendency toward the cytoplasm’s periphery. This effect was especially noticeable in cancer cells (Fig. 3 - D, F, H, J), where the β-tubulin presence was reduced in F-actin enriched areas of the cell periphery. The chondrosarcoma cells (Fig. 3 - B) exhibited a similar β-tubulin distribution to their healthy counterparts (Fig. 3 - A), with long, rich, well defined, and elongated microtubule networks, a feature shared by the rest of the healthy cells (Fig. 3 - E, G, I).

**Fig. 4:**
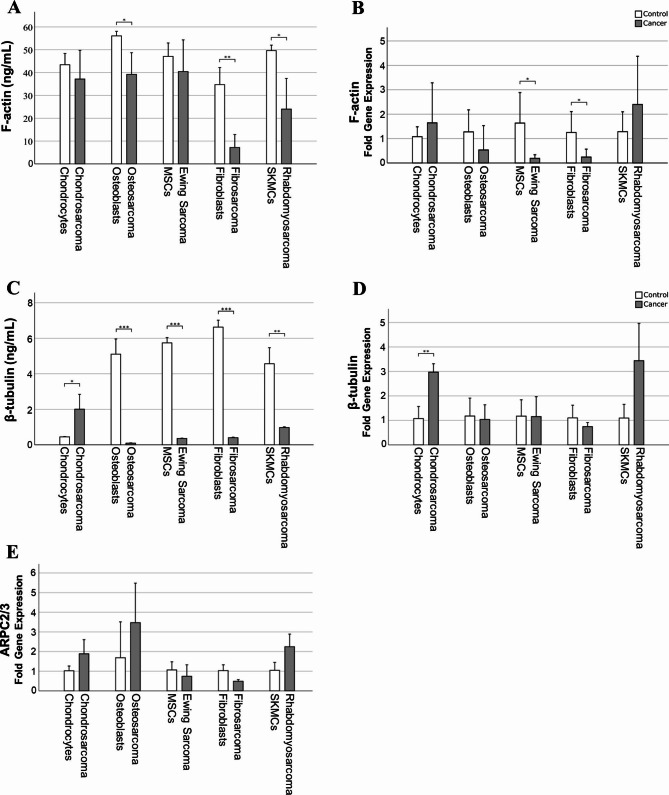
Quantification of cytoskeleton composition in musculoskeletal cancer lines and corresponding healthy cells. (**A**) F-actin protein (ELISA) and (**B**) gene expression (qPCR), (**C**) β-tubulin protein and (**D**) gene expression, (**E**) ARPC 2/3 complex gene expression. Data presented as bar diagram ± SD (n = 3). *p < 0.05, **p < 0.01, ***p < 0.001. Abbreviations: ARPC 2/3 - actin-related protein 2/3 complex, MSC- mesenchymal stem cells, SKMC - skeletal muscle cells

We further looked into the cytoskeleton composition to get a better understanding of the changes we saw at the structural level. To this end we quantitatively analyzed actin filaments (F-actin), microtubules (β-tubulin) and actin-related protein 2/3 (ARPC 2/3) complex known to act as a nucleus for actin polymerization (Fig. 4).

While there was a significant difference in F-actin protein content between osteoblasts and osteosarcoma (p = 0.020), fibroblasts and fibrosarcoma (p = 0.004), and SKMC and rhabdomyosarcoma (p = 0.016), no other cancer-healthy duo group showed a substantial difference in the F-actin protein content (Fig. 4 - A). At the gene-expression level, a similar trend was seen, between MSCs and Ewing sarcoma (p = 0.018) and fibrocytes and fibrosarcoma (p = 0.023, Fig. 4 - B).

The total protein content of β-tubulin was significantly lower in sarcoma cell lines compared to controls (osteoblasts – osteosarcoma, p = 0.001; MSC - Ewing sarcoma, p = 0.001, fibroblasts - fibrosarcoma p = 0.001; respectively SKMC – rhabdomyosarcoma, p = 0.002, Fig. 4 - C). In contrast, the opposite trend was observed for the chondrocyte – chondrosarcoma duo, with the chondrosarcoma cells exhibiting significantly more β-tubulin (p = 0.032). A similar trend was seen at the gene level, where β-tubulin expression was significantly upregulated in chondrosarcoma cells (p = 0.005), while the remaining difference between the other cancer-control duos did not reach statistical significance.

The ARPC2/3 expression (Fig. 4 - E) was tendentially elevated in cancer cells relative to their healthy counterparts, except for the fibroblasts-fibrosarcoma group in which the opposite trend was significantly observed (p = 0.038).

## Discussion

Due to the wide range of histological subtypes and clinical and histopathological characteristics that are not always distinct, musculoskeletal sarcomas remain a diagnosis challenge [27]. To date, there are still no reliable biomarkers that can be used for disease surveillance and screening. With the advent of quick biomechanical assaying techniques, stiffness may become a particularly important biomarker [28, 29] for load bearing tissues. In our study, we sought to determine whether cell stiffness is a valid, universal biomarker for different musculoskeletal sarcoma cell entities. Our study was designed as a large-scale exploratory one, in which we used the same AFM method and micro-mechanical indentation analysis on some of the major sarcoma entities to compare 10 cell lines, including 5 distinct sarcoma cell lines and 5 controls (healthy cells).

Our AFM results showed that, in four of our five experimental groups, the heathy cells were significantly stiffer than their corresponding cancer cell lines (osteosarcoma, Ewing sarcoma, fibrosarcoma, rhabdomyosarcoma). These results are in line with previous research showing reduced elastic moduli values of cancer cells compared to healthy cells [7, 8, 26]. Healthy and non-invasive cells are thought to have bulk stiffnesses far from the critical range, whereas cancerous and invasive cells are thought to modulate their stiffness to a value near the critical range in order to maximize the migratory potential required for tumor progression [30]. Softening tumor cells boost their ability to self-renew [31]. Although a correlation has been established between cell stiffness and tumor cell malignancy [32], the link between cellular mechanical properties and metastatic preference remains inconclusive.

One of the most interesting findings of our study was that the chondrosarcoma cell line had the exact opposite elastic fingerprint as the other sarcoma cell lines we looked at - it was much stiffer than the healthy control - chondrocytes. This interesting observation might be attributed to the molecular fingerprint of the neoplastic entity, and the nuanced differences between the cancer cell and the healthy control cell: chondrocytes. Chondrosarcomas have been shown to express several proteins known as MSC markers [33]. In fact, two cell types with distinct marker expression signatures have been isolated from primary conventional chondrosarcomas: one group of multipotent MSC origins (CD49b high/CD10 low/CD221 high), and another that resembled fibroblastic lineage (CD49b low/CD10 high/CD221 low) [34], suggesting that both chondrosarcoma cell types arose from multipotent MSC origins, the presumed origin of chondrosarcomas [34]. In fact, when looking at the elastic profile obtained in our study of the chondrosarcoma (median of 0.414 kPa) it resembles closely the MSC elastic profile (0.381 kPa) (p = 0.358). While adult chondrocytes also express MSC markers (CD105/CD166) [35] that increase with osteoarthritic driven degeneration of the articular cartilage [36], it is unclear whether this increase in MSC markers is an attempt at cartilage repair or a prerequisite for macroscopic cartilage degradation due to a lack of extracellular matrix maintenance. The differences between the two cell lines were also corroborated by a study done by Gabauer et al. which found only very limited similarities between SW1353 chondrosarcoma cells and chondrocytes, implying that the SW1353 cell line has a very limited potential to mimic chondrocytes [37].

Reorganization of the cytoskeleton, particularly actin microfilaments, has been shown to play a critical role in all aspects of cancer pathomechanism, including cell invasion and metastasis [38–40]. As such, we examined the organization of F-actin both qualitatively and quantitatively in these cell lines and compared their cytoskeletal architecture. All musculoskeletal cancer cells (chondrosarcoma, osteosarcoma, fibrosarcoma, Ewing sarcoma and rhabdomyosarcoma) exhibited a distinct F-actin arrangement pattern, barely displaying organized actin stress fibers (Fig. 3). This is consistent with previous research that also found actin stress fibers in the apical regions of healthy cells, but not in cancerous cells, suggesting that there fibers cannot contribute to stiffness [41]. This peculiar cytoskeletal reorganization might represent an adaptation mechanism used by the cancerous cells to adjust their stiffness to match the compliance of their substrates [42, 43]. The organization of the actin network can be altered by actin-binding proteins, among which is the ARPC2/3 that induces the formation of branched filaments, affecting actin dynamics [44]. Deregulation of the ARPC 2/3 regulatory framework has previously been described in cancer migration [45]. Our results showed that it is mainly expressed in cancer entities as well as in fibroblasts. The ARPC 2/3 complexes has been detected in both filopodia and lamellipodia of spreading fibroblasts, and their interplay is considered a significant factor in determining the motility choices made by the cells [46].

Also, in the healthy - cancer cell group where the cancer cells showed a lower stiffness (osteosarcoma, fibrosarcoma, Ewing sarcoma, and rhabdomyosarcoma), there was a decrease in the quantity of filamentous actin (F-actin) fibers, albeit to a lesser extent in the chondrosarcoma cell line (SW1335). These features reinforced the notion that the chondrosarcoma cell line has a distinct molecular, structural and mechanical fingerprint. In fact, previous research found an inverse relationship between the malignancy of chondrosarcoma cells and their degree of chondrocytic differentiation, implying, thus, that their metastatic ability was more dependent on the expression of specific matrix metalloproteases, that are required for egress from the tumor matrix and invasion into the extracellular matrix [47, 48]. Similarly a study done by Calzado-Martín et al. showed that although actin stress fibers contribute significantly to stiffness in healthy breast cells, the elasticity in tumorigenic cells does not appear to be primarily determined by these structures [41].

It is also conceivable that in chondrosarcomas, actin stress fibers are not the primary candidates responsible for changes in the stiffness profile. Interestingly, when looking at the cytoskeleton from the microtubules perspective, β-tubulin was significantly upregulated in this cell line (SW1335) when compared to chondrocytes (Fig. 4- C, D). The remaining cancer-healthy cell duos showed an exact opposite trend. Moreover, the beta-tubulin data seems to be consistent with the AFM stiffness data (Fig. 2), which showed that for all cancer cells-healthy controls duos, the healthy cells were significantly stiffer than the cancer cells, with the exception of chondrocytes-chondrosarcoma, where the opposite effect was observed. In fact, prior studies have demonstrated that microtubules possess a similar elastic modulus as actin filaments, measuring around 1 GPa, however, they also exhibit a bending rigidity approximately 100 times greater than that of actin [49, 50]. As dynamic components, microtubules also have the ability to form bundles with the aid of other proteins, consequently enhancing their stiffness. Remarkably, cross-linking two microtubules leads to a four-fold increase in stiffness, a considerable alteration that highlights their substantial role in cellular mechanics [51].

The presence of tumor cells with varying degrees of stiffness within the same tumor tissue may be due to the heterogeneity of the tumor mechanical microenvironment [52]. As a result, it is unclear whether the unique cell cytoskeleton arrangement, composition and stiffness are due to their adaptation to the particular mechanical microenvironment of the targeted organ or to their intrinsic features independent of extracellular factors. Also, it should be noted that because we only studied one chondrosarcoma cell line - SW1335, thus, it is highly conceivable that the elastic behavior varies between cell lines from the same cancer entity, as chondrosarcoma is known to be a highly heterogeneous disease [53]. Darling et al., showed that among 3 different grade II chondrosarcoma cells lines (JJ012, FS090, and 105KC) the mechanical properties differ significantly [54], which also exhibit different levels of aggressiveness and metastatic potential. The authors also suggested that cell deformability may reflect certain phenotypic characteristics associated with the metastatic process [54].

Overall, we found that most sarcoma cell lines from the musculoskeletal neoplastic family are associated with significant stiffness decrease at a cellular level coupled with a specific structural and compositional rearrangement of the cytoskeleton. Together with previous biochemical findings, these results could lead to new diagnostic or prognostic approaches at a cellular level for determining the metastatic potential of musculoskeletal sarcomas. Recent studies, for example, have reported a microfluidic cell sorting approach based on cell stiffness that can identify molecular mechanisms of drug resistance and examine the heterogeneous responses of cancers to therapies [55]. Moreover, a better understanding of the mechanisms underlying these mechanical changes coupled with tumorigenic transformation could lead to the development of new pharmacological approaches (i.e. inhibiting metastasis by chemically targeting cytoskeletal structures that regulate cell stiffness and its subsequent motility).

## Conclusion

This study suggests that changes in cellular stiffness are a peculiar feature found in the majority of musculoskeletal sarcomas investigated. The mechanical properties of chondrosarcoma cell lines show a distinct mechanical fingerprint, that potentially may vary substantially depending on the cell line tested. The structural rearrangement and composition of the cell cytoskeleton are also distinguishing features of these cell lines. These findings highlight the importance of cell stiffness in musculoskeletal sarcomas, which may not only reflect but also influence metastatic potential, and may be utilized for diagnostic, prognostic or therapeutic purposes.

### Study limitations

Cellular heterogeneity in tumors is a well-established phenomenon [56, 57] that is thought to be an important cause of drug resistance that impedes treatment outcome [58, 59]. Since, we only examined one patient-derived cell line for each neoplasia type in our study, the entire neoplastic cell population may not be represented. Our findings are, however, highly consistent with the previous publications that analyzed and showed a loss of stiffness in cancer cells coupled with a structural rearrangement of the cytoskeleton [8, 26, 30, 31]. It also has to be noted that our heathy control for the chondrosarcoma – the chondrocytes, were isolated from osteoarthritic cartilage samples received from patients receiving total knee replacement surgery. Thus, it is possible that, several cellular and molecular features of these cells may be altered when compared to chondrocytes derived from completely healthy cartilage samples.

## Data Availability

The dataset used and/or analysed during the current study are available from the correcponding author on reasonable request.

## List of abbreviations

AFM: Atomic force microscopy
BSA: Bovine serum albumin
DMEM: Dulbecco´s modified eagle medium
ECM: Extracellular matrix
ELISA: Enzyme-linked immunosorbent assay
EM: Elastic modulus
FCS: Fetal bovine serum
MSC: Mesenchymal stem cells
PBS: Phosphate buffered saline
PFA: Paraformaldehyde
qRT-PCR: Real-time quantitative polymerase chain reaction
SD: Standard deviation
SKMC: Skeletal muscle cells
